# Fast comparison of DNA sequences by oligonucleotide profiling

**DOI:** 10.1186/1756-0500-1-5

**Published:** 2008-02-28

**Authors:** Vicente Arnau, Miguel Gallach, Ignacio Marín

**Affiliations:** 1 Departamento de Informática. Universidad de Valencia, Spain; 2 Departamento de Genética. Universidad de Valencia, Spain; 3 Instituto de Biomedicina de Valencia. Consejo Superior de Investigaciones Científicas (IBV-CSIC), Spain

## Abstract

**Background:**

The comparison of DNA sequences is a traditional problem in genomics and bioinformatics. Many new opportunities emerge due to the improvement of personal computers, allowing the implementation of novel strategies of analysis.

**Findings:**

We describe a new program, called UVWORD, which determines the number of times that each DNA word present in a sequence (target) is found in a second sequence (source), a procedure that we have called oligonucleotide profiling. On a standard computer, the user may search for words of a size ranging from *k *= 1 to *k *= 14 nucleotides. Average counts for groups of contiguous words may also be established. The rate of analysis on standard computers is from 3.4 (*k *= 14) to 16 millions of words per second (1 ≤ *k *≤ 8). This makes feasible the fast screening of even the longest known DNA molecules.

**Discussion:**

We show that the combination of the ability of analyzing words of relatively long size, which occur very rarely by chance, and the fast speed of the program allows to perform novel types of screenings, complementary to those provided by standard programs such as BLAST. This method can be used to determine oligonucleotide content, to characterize the distribution of repetitive sequences in chromosomes, to determine the evolutionary conservation of sequences in different species, to establish regions of similar DNA among chromosomes or genomes, etc.

## Findings

There are a few qualitatively different types of analyses of DNA sequences. First, we find methods to detect similarity, often to generate pairwise or multiple alignments (e. g. those implemented in BLAST, CLUSTALX, etc.). A second type of analysis is dedicated to discover patterns of conserved motifs in multiple sequences (e. g. MEME). A third characteristic class includes the programs implementing phylogenetic analyses of DNA data (e. g. MEGA4, PAUP). Finally, a fourth significant class involves alignment-free sequence comparisons (reviewed in [1]). Many of the methods included in this fourth class depend on the analysis of the frequencies of different "words" of nucleotides. Word analysis has contributed to determine fundamental aspects in genomics, such as compositional biases among chromosomes or genomes, asymmetries between the strands of the double helix, biases in codon usage, patterns of DNA methylation diminishing CG dinucleotides, discovery of binding sites for transcription factors, etc. (reviewed in [2]). It is thus of great interest to have fast, flexible tools for exhaustive exploration of DNA words at a genomic scale. A problem of this type of analysis is how to generate algorithms able to compile and store the information for the large amounts of different words arising when large values of *k*, the word length, are used. One solution is to use complex preprocessing of the data and then fast multiprocessor machines, which allow for exhaustive explorations of words of any size at a genomic scale (e. g. [3-5]). These approaches have the obvious drawbacks that not all potential users may have access to parallel equipment. Moreover, each platform requires adjustments of the programs [5]. In fact, most users interested in word genome analysis would benefit from programs able to rapidly scan for relatively short words on standard computer equipment. Studies that exhaustively characterize words in chromosomes or full genomes generally search for sequences of sizes 1 ≤ *k *≤ 6 (e. g. [6-9]). Studies that look for all words of longer sizes are scarce (e. g. [10-14], for words up to *k *= 11). Only analyses focused on the detection of one or a few related sequences, binding sites for transcription factors or regulatory elements upstream of the genes, explore even longer words, generally up to 15 nucleotides long (e. g. refs. [6,15-18]).

### Oligonucleotide profiling using UVWORD

Here we describe a new program, UVWORD, which implements a strategy of analysis that we have called *oligonucleotide profiling*. It consists in establishing the frequencies in which all the oligonucleotides detected in a particular sequence ("target sequence") are present in a second sequence ("source sequence"). The method is as follows: UVWORD first searches for words of size 1 ≤ *k *≤ 14 present in the source sequence and determines their frequencies by using a sliding-window approach, moving one nucleotide in each step. Then, the program reads all words present in the target sequence. Finally, it associates each of the words in the target sequence with their corresponding frequencies in the source sequence. The user may ask the program to add together the frequencies for a number of adjacent positions in the target sequence. This is implemented in a a parameter that we have called *range *(*R*). The *R *value allows the user to choose between "fine grain" (typically *R *= 1; i. e. individual counts) and broad regional comparisons. For the latter, *R *values up to 10^5 ^– 10^6 ^(i. e. counts for 10^5 ^or 10^6 ^adjacent words) may be used. This is convenient when the target sequences are very long (see below). The program works at extremely fast speeds: from 3.4 (*k *= 14) to 16 (1 ≤ *k *≤ 8) millions of words per second on a PC computer with a 2.8 GHz Intel Pentium 4 processor and 2 Gb RAM.

We became interested in developing this program when we noticed that oligonucleotide profiling allows to perform types of analysis that are conceptually very different (Table 1). First, if we use a single sequence as both source and target, UVWORD will simply provide the user with the frequency of all the words present in that sequence. In this case, UVWORD is equivalent to other programs used to just count oligonucleotides. However, source and target sequences may be different and that allows for several interesting alternative analyses that cannot be performed with related programs. For example, if the source is large (e. g. whole chromosomes or even whole genomes) while the target is a short motif, UVWORD will provide the frequency in the long source sequence of all the words present in the motif. This may be useful to determine the degree of repetition in a chromosome of the words that compose a particular short sequence (e. g. to detect patterns of internal repeats in a satellite sequence, evolutionary conservation of sequences, etc). On the other hand, the inverse analysis (i.e. the source is a short motif and the target is a chromosome) allows, if the target sequence is divided into parts, to determine the distribution of the sequences present in the motif along the chromosome. Two large molecules can also be compared and their degree of general similarity can be established, both at a local scale and globally (see details in Table 1). As we will show in some biological examples, and it is also summarized in Table 1, most of these analyses cannot be performed unless words of sufficiently large size (typically *k *= 10), which generate sequences expected to occur very rarely by chance, are used.

**Table 1:** Some uses of the oligonucleotide profiling strategy. Typical values for the word size (*k*) and range parameter (*R*) for analyses involving eukaryotic chromosomes are detailed. If small eukaryotic chromosomes or bacterial genomes are analyzed, the most convenient *k *and *R *values may be smaller. When two or more sources are used, results are obtained independently and then compared. Some examples are shown in detail in the supplementary information (Supplementary figures 1 – 5).

**Type of analysis**	**Source**	**Target**	**Typical word sizes *(K)***	**Typical ranges *(R)***	**Examples**
Oligonucleotide, microsatellite quantification, chaos game representation	Any DNA sequence	Same as Source	1–8	1	See Refs. [2,21]
Degree of conservation within a repetitive sequence	Chromosome	Repetitive sequence	10–14	1	Suppl. Figs. 1A, 2
Variations in repetitive content	Two or more chromosomes	Repetitive sequence	10–14	1	Suppl. Fig. 3
Sequence localization	Short sequence	Chromosome	1–14	10^3^-10^6^	Suppl. Figs. 1B, 4
Degree of sequence conservation or changes in sequence complexity among chromosomes	Two or more chromosomes	One of the chromosomes	12–14	1–10^5^	Suppl. Fig. 5
Detection of singular sequences	Two chromosomes	One of the chromosomes	12–14	1	See Ref. [19]

UVWORD was written in C and it is compiled for Microsoft Windows and Linux operating systems. Its algorithm is very simple. First, a word of size *k *is read from the source sequence and the program computes for that word a hash value: each of the nucleotides in a word is converted using a two-bits binary code (A = 00, C = 01, G = 10, T = 11) into a number. Each particular word has thus an associated binary number or its corresponding decimal number. Consequently, 4^k ^different decimal numbers serve to represent all possible nucleotide sequences of size *k*. These decimal numbers are used as pointers to address a table of frequencies, in which a counter increases when a particular DNA word is found. This process is sequentially repeated for each nucleotide, until the source sequence is fully read in its 5' – 3' direction. After the source is analyzed, the program reads each word in the target file and searches for those words in the table of frequencies derived from the source sequence. UVWORD may exhaustively analyze words of size 1 ≤ *k *≤ 14 on a PC computer with at least 1.25 Gb RAM, or 1 ≤ *k *≤ 13, with 512 Mb RAM.

In order to use UVWORD, the sequences must be written in two standard text (.txt) or fasta (.fa) format files. Any comments or symbols other than A, C, G, T will be properly detected and skipped by the program. The program requires only two parameters, the word size *k *and the range, *R *(see above). Using these parameters, the program generates the results and writes them into a file (.out) of columns separated by tabs, which can be readily imported to other programs for further analysis or graphical representation.

### Biological examples

We have generated a few selected examples, described in detail in the supplementary information of this article [see Additional file 1]. They include 1) Characterization of the structure and location of X-specific satellites on the *Drosophila melanogaster *X chromosome; 2) Conservation of words in Alu repetitive sequences in human and chimpanzee; 3) Relative frequencies of Alu sequences in human and chimpanzee; 4) Distribution of CG dinucleotides, Alu and LINE1 elements in human and chimpanzee chromosomes; and, 5) Comparison of general profiles for human chromosomes 21 and 22 (details in Supplementary figures 1 – 5 [see Additional file 1]). A first paper of our group using this methodology has been recently published [19].

## Discussion and conclusion

It is often overlooked that the improvement of computer equipment confers well-known "brute force" methods the ability of providing qualitatively new types of information. The results that we have shown are good examples of how the extension of a classical type of analysis, which involves counting short words in DNA sequences, may be used in novel contexts. Here, that extension depends on two novel features. The main novelty in our approach is what characterizes the oligonucleotide profiling strategy: data from two sequences, one that provides the words to be analyzed (target) and a second sequence in which the number of times that those words are present is counted (source), are combined. The second significant feature is that most of the interesting analyses depend on the ability of exhaustively count all the words of size *k *= 10 in very long DNA sequences (see Table 1). This would have been a daunting task for a personal computer just a few years ago. Now, we routinely use *k *= 13 for most of these searches. There are two reasons for choosing this particular word size, especially to analyze long chromosomes. First, sequences of 13 nucleotides are already extremely specific. In a random sequence, we expect to find each word of size *k *= 13 just once every 67 millions of words. This means that if we search for a particular 13-mer, characteristic of a given sequence, in even the longest eukaryotic chromosomes, the number of false positives – sequences that will be identical by chance to the one that we are looking for – is expected to be very low. The second reason to prefer *k *= 13 to other sizes such as *k *= 12 or even *k *= 14, which can also be used with our current version of UVWORD, is that 13 is a prime number. This fact contributes to avoiding systematic patterns that may increase the noise, associated to the presence above expectation of particular dinucleotides, trinucleotides (some typically enriched in coding regions), etc.

The information that can be extracted from an UVWORD output is often more precise or useful than the mere establishment of similarity or the localization of sequences similar to a query that can be distilled from the output of a BLAST search. In fact, oligonucleotide searches and BLAST searches are complementary. For example, BLAST searches allow for a fast quantification of the number and localization of repetitive sequences and, by the fact that mismatches are allowed in the detection of similarity, they are clearly superior to UVWORD searches unless very short, identical oligonucleotides are sought. However, oligonucleotide profiling is clearly superior for establishing the degree of conservation in repetitive sequences (e. g. Supplementary figures 1A, 2 [see Additional file 1]), which would be very arduous to infer from BLAST searches. It is also clearly superior to establish the patterns of global similarity among chromosomes (Supplementary figures 4, 5 [see Additional file 1]), that cannot be so readily explored using BLAST. The detection of singular sequences or patterns is also simpler using UVWORD (e. g. Supplementary figure 3 [see Additional file 1]).

In summary, we think that the oligonucleotide profiling strategy implemented in UVWORD can be useful to all researchers interested in exploring nucleotide sequences for significant patterns. Our program has, in addition of its versatility, all the advantages that we may ask before deciding to add a new program to our arsenal: it does not require additional, expensive computer equipment, it can cope with the largest available sequences, it is very fast and it is extremely simple to use. Its simplicity allows modifications of UVWORD for particular uses to be tailor-made quite easily. For instance, we developed a version focused on the automatic determination of sequences that were very frequent in a chromosome and absent in another chromosome [20]. The program can also be easily modified to perform related tasks, for example, to generate chaos game representation of sequences [10,13,21].

## Availability and requirements

Project name: UVWORD

Project home page: 

Operating systems: Windows and Linux versions available

Programming language: C

Other requirements: none

License: UVWORD versions for Windows and Linux (32- and 64-bit processors) can be downloaded from . It is free for academic users, no license required.

Any restrictions to use by non-academics: it requires to sign a license agreement.

## Competing interests

The authors declare that they have no competing interests.

## Authors' contributions

VA and IM devised the oligonucleotide profiling strategy. In addition, VA wrote the UVWORD program while IM, made a few analyses, coordinated the research and wrote the manuscript. MG performed most of the analyses shown here and also provided many useful ideas for the development of UVWORD.

## Supplementary Material

Additional file 1 A summary of biological examples can be found in the Supplementary information of this paper file Click here for file
