# Supplementary material for: Fast comparison of DNA sequences by oligonucleotide profiling
Source: BMC Res Notes. 2008 Feb 28;1:5. doi: 10.1186/1756-0500-1-5 (PMC2518268; doi:10.1186/1756-0500-1-5)

Representative oligonucleotide profiling-based analyses

They are summarized in Supplementary Figures 1 to 5. Supplementary figure 1 shows analyses involving *Drosophila melanogaster* sequences. In Supplementary figure 1A, a 1350 bp-long sequence corresponding to most of the *Drosophila melanogaster* X chromosome-specific satellite described by DiBartolomeis et al. (ref. [1] in the Supplementary reference list below; Accession number X62937) was used as target sequence while the source sequence was the *D. melanogaster* X chromosome. The graph corresponds to the frequencies of each word (k = 13) found in the satellite. This high value of k was chosen to avoid that non-satellite sequences are counted. In a chromosome of this size and with k = 13, the expected number of sequences that by chance would be identical to those in the satellite was about 0.3 (i. e. negligible). In Supplementary figure 1A, it is evident the internally repetitive structure of this satellite which explains the four main peaks found along the sequence, each one corresponding to sequences present more than 100 times in the X chromosome. Supplementary figure 1B shows the inverse analysis. When the chromosome X is used as target and the source is the most frequently repeated 13-nucleotide long sequence found in the satellite (CAAATTTTGATGA), we can determine the distribution of that particular sequence along the chromosome. In Supplementary figure 1B, a range $R = 10^5$ is used to count the frequency of the word in 100 Kb intervals. In agreement with DiBartolomeis et al. data [1], the satellite sequence is found disperse in multiple places, with only one region concentrating a considerable number of copies. Remarkably, the satellite is not present in positions close to the tips of the chromosome. The two analyses shown in Supplementary figures 1A and 1B thus demonstrate that oligonucleotide profiling may be used to characterize the internally repetitious structure of a sequence (Supplementary

figure 1A) or to determine the distribution of a particular significant sequence in a long chromosome (Supplementary figure 1B), provided that *k* is sufficiently large.

Supplementary figure 2 shows a comparison of the frequencies of words typical of the highly repetitive Alu elements in human and chimpanzee chromosomes. The first, highly conserved, 153 nucleotides of a consensus AluY sequence (obtained from [2]; p. 725) were used as target and the sources were two homologous chromosomes: human chromosome 21 and chimpanzee chromosome 22 [3-4]. Again we used $k = 13$ to avoid noise caused by random sequences. In this case, the expected number of sequences to be spuriously counted in each analysis was about 0.5, again irrelevant for obtaining accurate conclusions. As it can be seen in Supplementary figure 2, almost identical conservation pattern are observed in both species. The highest peaks correspond to the most conserved sequences within the Alu element, which lack CG dinucleotides (see asterisks at the bottom of the Supplementary figure). This is caused by methylation of CG dinucleotides generating a fast rate of mutation, and thus leading to diversification of Alu sequences, in such a way that most of them are not identical to the consensus [5]. This type of oligonucleotide profiling thus allows for the establishment of the degree of constraint acting on repetitive sequences both within a genome and between genomes.

Supplementary figure 3 shows the differences in number of Alu sequences in human versus chimpanzee, expressed as the ratio [human Alu sequences / chimpanzee Alu sequences], and corrected for the size of both chromosomes. For the same reasons that in the previous examples, $k = 13$ was used. Here, the target sequence was a consensus human Yb8 Alu sequence (Acc. No. AC093768) and the sources were the two primate chromosomes. Most ratios, especially those that correspond to sequences

that are common to all Alu subfamilies (see region corresponding to Supplementary figure 2, on the left), are close to 1. This demonstrates that general divergence in Alu sequences since both primate lineages diverged has been small. However, there are several striking differences. Particularly, a region specific of the Alu Yb8 subfamily sequences (see ref. [2] for a summary) and absent in other Alu sequences, is found only in the human chromosome (Supplementary figure 3). This is due to the fact that consensus Alu Yb8 sequences are very frequent in humans (2200 elements; [2]), while are very rare in chimpanzees (9 sequences detected by those same authors). These results thus demonstrate that Alu Yb8 sequences are absent in chimpanzee chromosome 22, while they are relatively frequent in human chromosome 21. This is a typical example of how subtle interspecific singularities in sequence conservation can be detected by oligonucleotide profiling.

In Supplementary figure 4, we show a summary of the UVWORD results when human and chimpanzee chromosomes are scanned for general features, such as frequency of Alu sequences, LINE1 sequences (a conserved, 13-nucleotide long sequence obtained from LINE1 reverse transcriptase was used: TCAGGATACAAAA; accession number L19088.1) or CG dinucleotide repeats. The Supplementary figure demonstrates the extreme similarity of the profiles of human and chimpanzee chromosomes. It is easy to see that this assembly of the chimpanzee chromosome is lacking part of the sequences that would correspond to the 5’ end of the human chromosome, but otherwise results are almost indistinguishable. This example shows how oligonucleotide profiling can be used not only to quantify particular sequences but also to characterize the global patterns of sequence similarity among chromosomes.

Finally, in Supplementary figure 5 we show one of the results that can be obtained from a more detailed, quantitative, comparison of two eukaryotic chromosomes. Sequences from human chromosome 22 and from two regions characteristic of human chromosome 21, one of them gene-rich and the other gene-poor, are compared. In Supplementary figures 5A and 5B, the target was a set of 500000 words randomly extracted from a 18.0 Mb-long sequence that correspond to part of the gene-rich region of chromosome 21 (Mb 12-30 in the map described in [3]; gene density: 6.6 genes/Mb). When we use as source the region from which the sequences have been obtained, we obtain frequencies that we represent in the x-axis. In the y-axis, we represent the frequencies of those same sequences, but when we use as source either 35.1 Mb corresponding to all the available sequences for the chromosome 22 (Supplementary figure 5A; gene density: 21 genes/Mb) or a region of 7.0 Mb extracted from the longest gene-poor region of the chromosome 21 (Supplementary figure 5B; region 5-12 Mb in the map by Hattori et al. [3]; density: <1 gene/Mb). If the nucleotide composition was identical in the two sources, and once corrected the effect caused by using sources of different sizes, we would expect to find each sequence in equal frequency. These expected results are summarized by the dotted lines in Supplementary figure 5. However, as we can see in Supplementary figures 5A and 5B, substantial differences from the expected values were observed. When the gene-rich region of chromosome 21 was compared with the chromosome 22 (Supplementary figure 5A), we observed a slope (continuous line) that is steepest than expected by chance. On the contrary, when we compared the gene-rich region of chromosome 21 with the gene-poor region of the same chromosome (Supplementary figure 5B), the slope, again shown by a continuous line, was less steep than expected by chance. This effect is caused by differences in the complexity of these sequences. The gene-rich region of chromosome 21 is much more

repetitious, less complex, than the gene-poor region of chromosome 21, but more complex than chromosome 22. It is clear inspecting Supplementary figure 5A that the effect of the different amounts of repetitive sequences is more important (see cloud of points around the continuous line) than the effect of having sequences that are unique or more abundant in the sequence used as target, which generate points below the expected, dotted lines. The fact that the chromosome 21 gene-poor region is the more complex of the three is confirmed by results shown in Supplementary figure 5C and 5D, in which 500000 words were again randomly taken, but this time from that gene-poor region. Comparisons of the different sources demonstrates that, on average, those words are more often present in the gene-rich region of the chromosome 21 or in the chromosome 22 that in the gene-poor region from which they derive. Taken all together, these results may seem counterintuitive, because they show that, in these chromosomes, complexity inversely correlates with gene density, in spite of the inherent high degree of complexity of coding sequences. The reason for this situation is the large increase in Alu repetitive sequences associated with the gene-rich regions in these two chromosomes [3, 6]. The increment in Alu sequences hides the expected positive correlation between gene density and DNA complexity. This final example demonstrates the power of oligonucleotide profiling to characterize global quantitative differences among very large DNA sequences.

Figure legends

Supplementary figure 1. Two typical uses of UVWORD. A) Characterization of the internal repetitive structure of a *Drosophila* X chromosome-specific satellite. Words of size k = 13 and a range R = 1 were used. B) Distribution of the satellite on the *Drosophila* X chromosome. In this last analysis, we used a range $R = 10^5$ (i. e. results refer to 100 Kb). See text for details.

Supplementary figure 2. Frequencies of 13-nucleotide-long DNA words found in an AluYb8 sequence (Acc. No. AC093768) in primate chromosomes. Black bars correspond to human chromosome 21 and grey bars to chimpanzee chromosome 22. Words that contain one or several CG dinucleotides are marked with an asterisk. R = 1 was used. A total of 153 nucleotides, and therefore 141 words, were analyzed.

Supplementary figure 3. Relative conservation of Alu sequences in human and chimpanzee chromosomes. The ratio [frequency of 13-mers in human] / frequency of 13-mers in chimpanzee] was corrected for the relative chromosome sizes to generate a distribution with expected values = 1 (white line), assuming no differences in both species. The sequence detailed on the top was not found in chimpanzee chromosome 22 (ratio = ∞). The highly conserved region used in the analyses shown in Figure 2 is also pinpointed (dashed line).

Supplementary figure 4. Chromosomal profiles. Distribution of Alu and LINE1 elements and of CG dinucleotides along human and chimpanzee chromosomes. Parameters for Alu and LINE1: k = 13; $R = 2\ 10^4$. Parameters for CG dinucleotides: k = 2; $R = 2\ 10^4$.

Supplementary figure 5. Comparisons of the general oligonucleotide profiles of human chromosome 22 (Hs22) and two regions of human chromosome 21 (Hs21), one of them rich in genes and the second gene-poor. Dashes lines correspond to the expected values once results are corrected by the sizes of the three sequences. Continuous lines show the slope of the linear regression of the observed data. A few

values that were present in frequencies higher than 1000 are not shown, although they were considered to calculate the regression values. See details in the text.

Supplementary references

1. DiBartolomeis SM, Tartof KD, Jackson FR: **A superfamily of *Drosophila* satellite related (SR) DNA repeats restricted to the X chromosome euchromatin.** *Nucl Acids Res* 1992, **20:**1113-1116.

2. Gibbons R, Dugaiczyk LJ, Girke T, Duistermars B, Zielinski R, Dugaiczyk A: **Distinguishing humans from great apes with AluYb8 repeats.** *J Mol Biol* 2004, **339:**721-729.

3. Hattori M, Fujiyama A, Taylor TD, Watanabe H, Yada T, Park H-T, Toyoda A, Ishii K, Totoki Y, Choi D-K et al.: **The DNA sequence of human chromosome 21.** *Nature* 2000, **405:**311-319.

4. International Chimpanzee Chromosome 22 consortium: **DNA sequence and comparative analysis of chimpanzee chromosome 22.** *Nature* 2004, **429:**382-388.

5. Xing J, Hedges DJ, Han K, Wang H, Cordaux R, Batzer MA: **Alu element mutation spectra: molecular clocks and the effect of DNA methylation.** *J Mol Biol*, 2004, **344:**675-682.

6. Dunham I, Shimizu N, Roe BA, Chissoe S, Hunt AR, Collins JE, Bruskiewich R, Beare DM, Clamp M, Smink LJ, et al.: **The DNA sequence of human chromosome 22.** *Nature* 1999, **402**:489-495.

Supplementary Figure 1. Arnau et al.

A

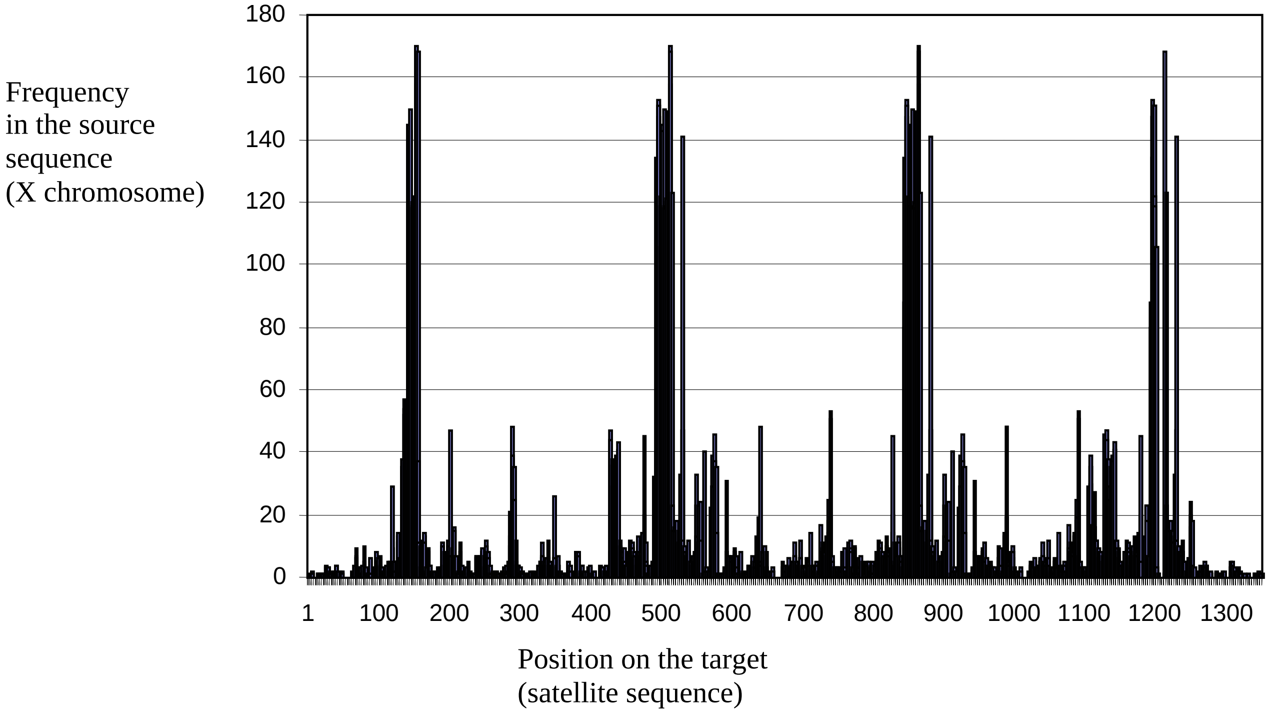

B

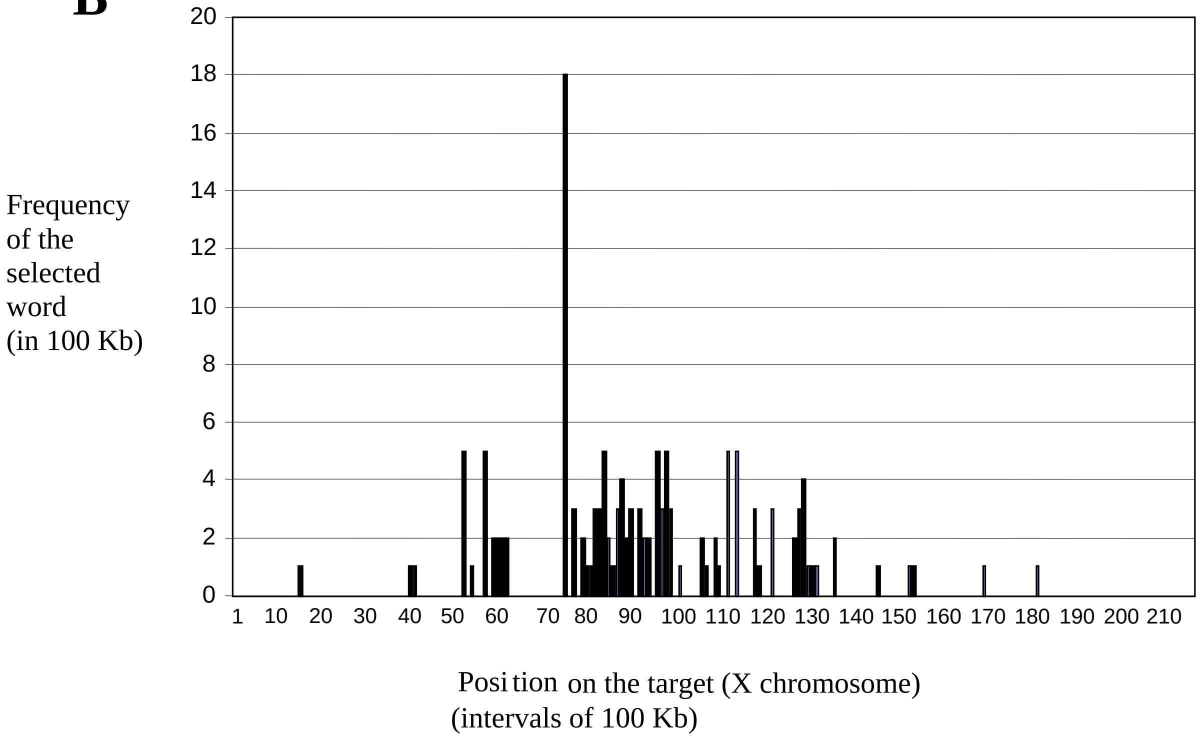

Supplementary figure 2. Arnau et al.

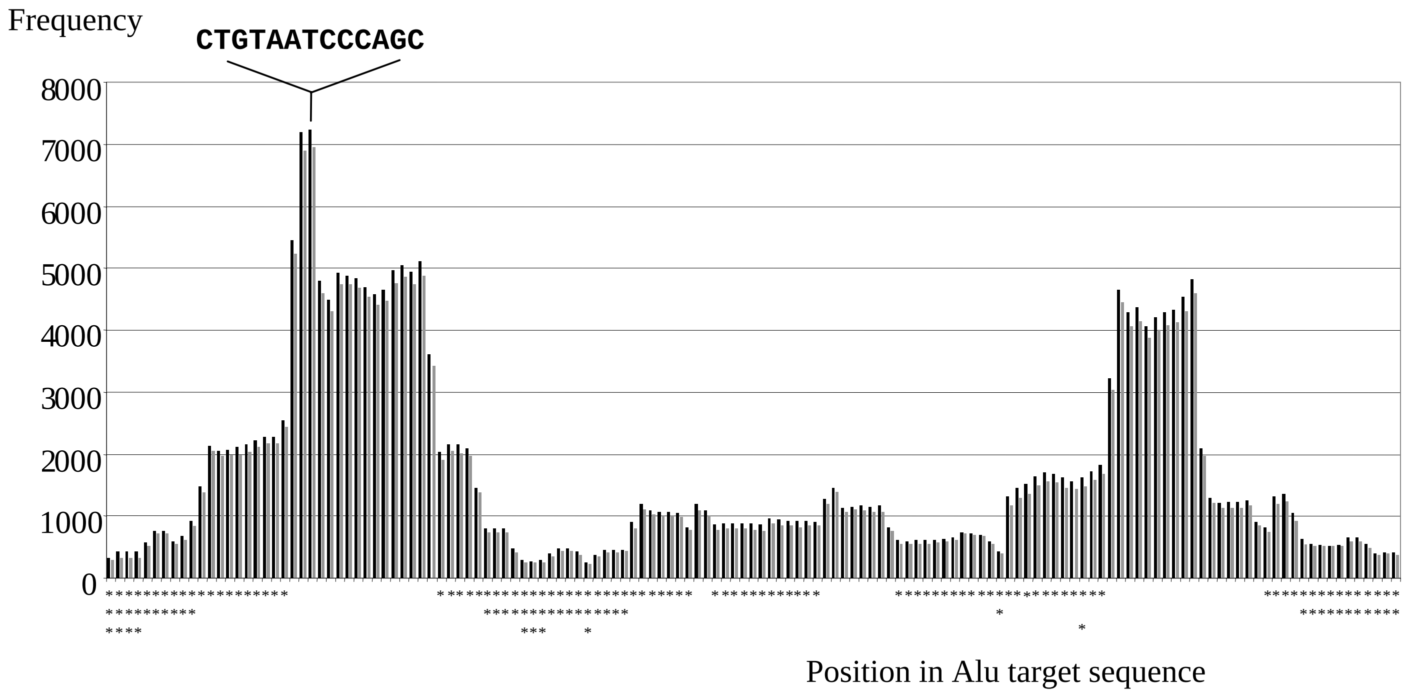

Supplementary Figure 3. Arnau et al.

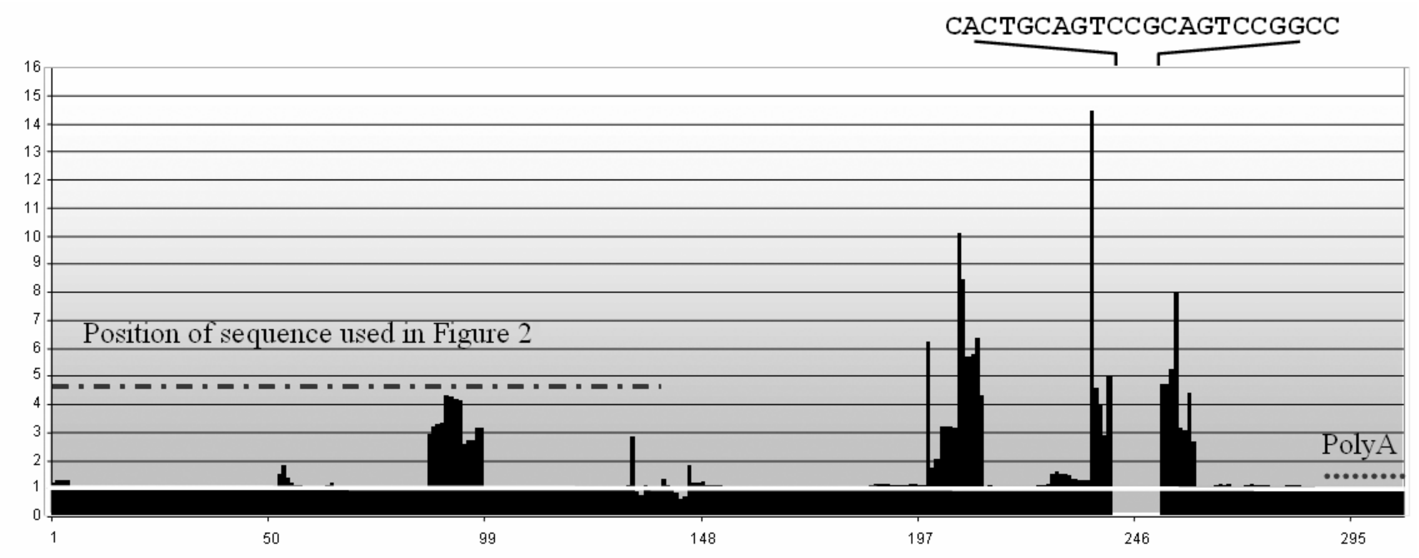

Supplementary figure 4. Arnau et al.

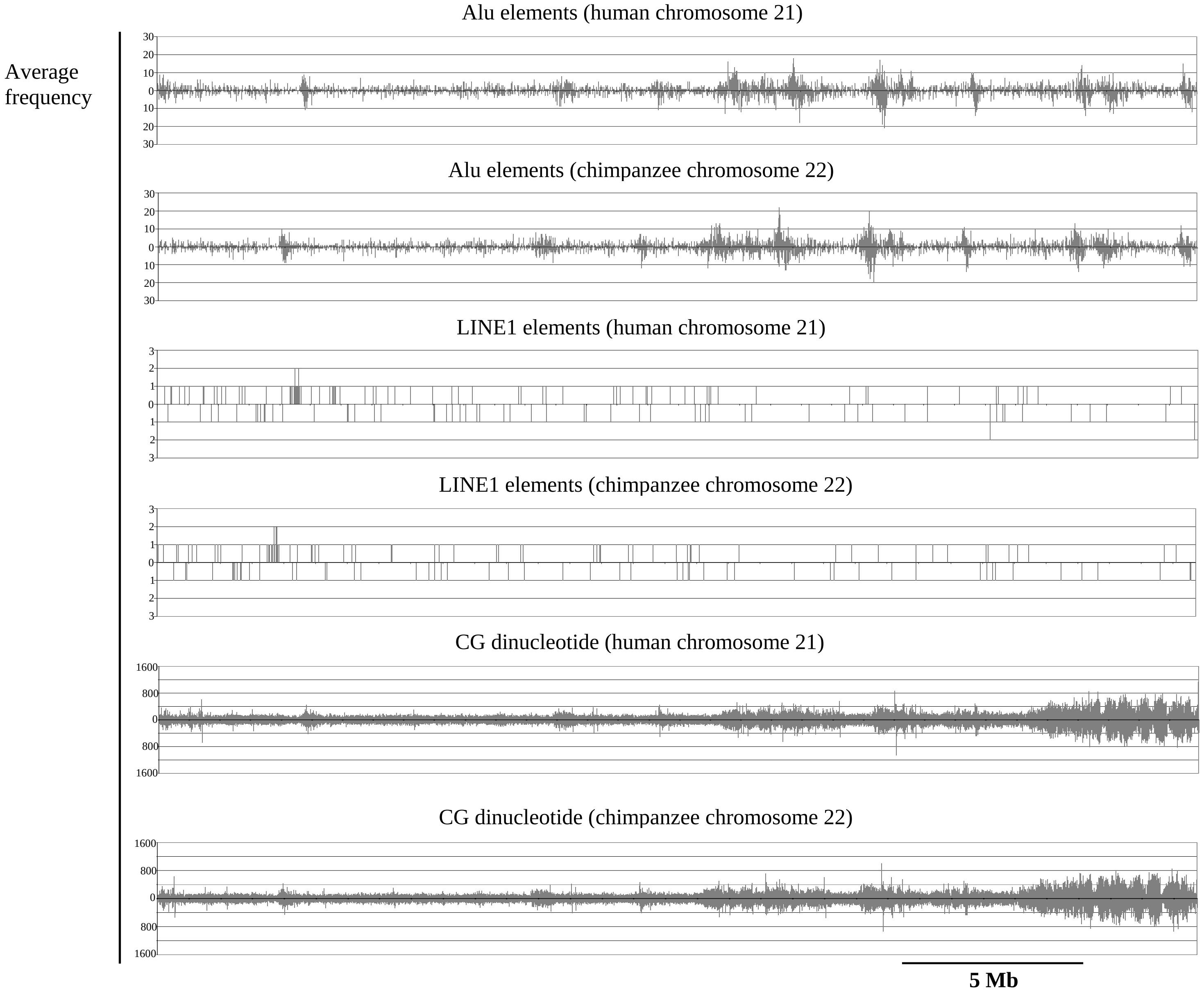

Supplementary figure 5. Arnau et al.

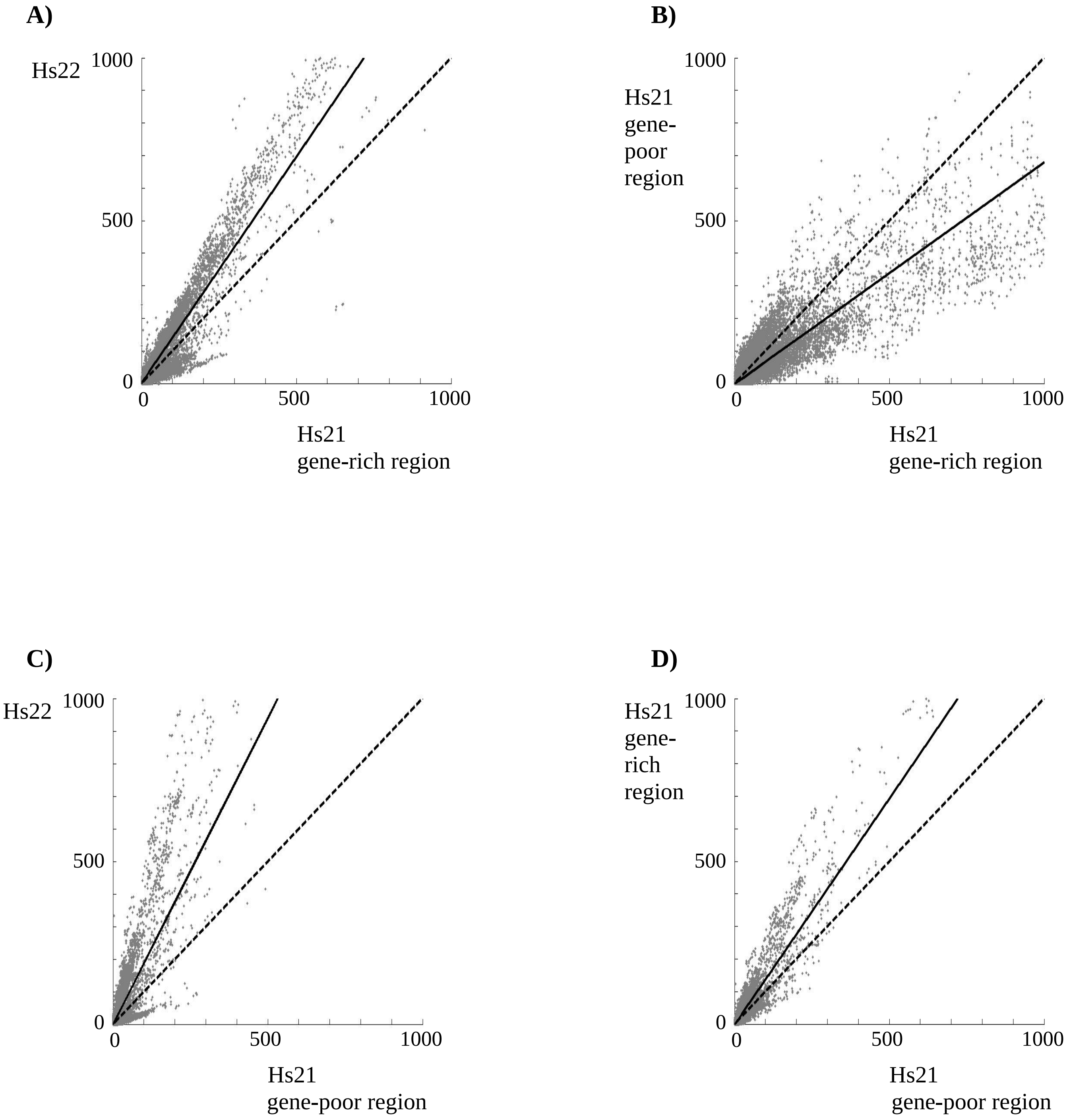

Supplement: Additional file 1 — A summary of biological examples can be found in the Supplementary information of this paper file [file 1756-0500-1-5-S1.pdf]
